# Peptide Receptor‐Functionalized AuNP‐Embedded Zwitterionic Biopolymeric Nanohydrogel for Electrochemical Sclerostin Sensing

**DOI:** 10.1002/advs.74575

**Published:** 2026-02-25

**Authors:** Hyo Jeong Yang, Jae Hwan Shin, Mingi Jo, Changyeon Lee, Hyunmin Yi, Al‐Montaser Bellah H. Ali, Jong Pil Park

**Affiliations:** ^1^ Department of Food Science and Technology and GreenTech‐Based Food Safety Research Group BK21 Four Chung‐Ang University Anseong Republic of Korea; ^2^ Department of Chemical Engineering Chung‐Ang University Seoul Republic of Korea; ^3^ Department of Chemical and Biological Engineering Tufts University Medford Massachusetts United States; ^4^ Department of Pharmaceutical Analytical Chemistry Faculty of Pharmacy Assiut University Assiut Egypt

**Keywords:** affinity peptide, chronic kidney disease, nanohydrogel, osteoporosis, sclerostin, zwitterionic polymer

## Abstract

Sclerostin (SOST) is a key negative regulator of bone formation and an emerging biomarker associated not only with osteoporosis but also with chronic kidney disease. Despite its growing clinical relevance, biosensing platforms for SOST remain limited, with most existing approaches relying on antibody‐ or aptamer‐based assays that often suffer from high cost, limited stability, and reduced performance in complex biological samples. Here, we report a peptide‐based electrochemical biosensing platform enabled by a multifunctional zwitterionic biopolymeric nanohydrogel, composed of chitosan, carboxybetaine methacrylate, and gold nanoparticles (CS–ZI–AuNPs). This nanohydrogel acts as a unified antifouling biointerface, simultaneously providing resistance to nonspecific adsorption, stable peptide receptor immobilization, and efficient electrochemical signal transduction. The resulting biosensor achieves picomolar sensitivity with a detection limit of approximately 26.4 pg/mL, enabling accurate quantification of SOST across clinically relevant concentration ranges. Importantly, the platform demonstrates statistically significant discrimination between healthy individuals and patients with osteoporosis, postmenopausal osteoporosis, and chronic kidney disease (stages 1 and 3) using real clinical samples. By integrating zwitterionic antifouling chemistry with peptide‐driven molecular recognition in a conductive nanostructured matrix, this work establishes a generalizable design principle for electrochemical biosensors targeting protein biomarkers. The proposed strategy offers a robust and scalable alternative to conventional immunoassays, with broad implications for translational diagnostics and real‐time disease monitoring.

## Graphical abstract

We report an electrochemical biosensor that employs biopanning‐derived affinity peptides as selective receptors on a multifunctional chitosan–zwitterionic–gold nanoparticle (CS–ZI–AuNPs) nanohydrogel interface. The CS–ZI–AuNPs nanohydrogel exhibits antifouling properties, abundant sites for stable peptide immobilization, and efficient electrochemical signal transduction, thereby integrating molecular specificity with signal amplification. This EC biosensor discriminates between healthy individuals and patients with osteoporosis, postmenopausal osteoporosis, and early‐stage chronic kidney disease, demonstrating its diagnostic relevance.

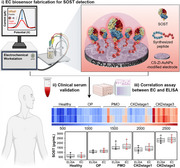

## 1 Introduction

As the global population ages, the importance of detecting biomarkers associated with bone‐related diseases has become increasingly evident. Sclerostin (SOST), a glycoprotein secreted primarily by osteocytes, is a key negative regulator of bone formation through inhibition of the canonical Wnt/β‐catenin signaling pathway [1]. This signaling cascade promotes osteoblast maturation and differentiation while upregulating osteoprotegerin expression in osteoblasts and osteocytes, thereby suppressing osteoclast formation and activity [2]. Elevated circulating levels of SOST have traditionally been recognized as a hallmark of osteoporosis (OP) and other low‐bone‐turnover disorders [3]. Furthermore, emerging evidence indicates that SOST is also dysregulated in chronic kidney disease (CKD), where impaired renal clearance and ectopic expression in vascular tissues contribute to its systemic accumulation [4]. This dual involvement in skeletal and renal pathophysiology underscores the potential of SOST as a systemic biomarker within the bone–kidney–vascular axis [5].

Despite its clinical significance, current detection paradigms for SOST primarily include immunoassays and emerging aptamer‐based biosensors [6]. Lateral flow immunoassays have enabled more rapid point‐of‐care testing, while conventional enzyme‐linked immunosorbent assays (ELISAs) provide acceptable sensitivity and specificity [7]. However, ELISAs remain time‐consuming and reliant on centralized laboratory facilities and are further limited by lengthy protocols, dependence on specialized infrastructure, and potential variability across commercial kits and biological matrices [8, 9]. Recent studies have explored aptamer‐based approaches, including bone‐targeting aptamer‐functionalized nanoparticles for the in situ modulation of SOST in OP therapy, as well as therapeutic aptamers targeting specific regions such as the Loop‐3 domain of SOST to stimulate bone formation without elevating cardiovascular risk [10, 11]. Although aptamers exhibit intrinsically high affinity and specificity, their performance in biological fluids can be compromised by nonspecific adsorption and matrix effects. More fundamentally, their highly polyanionic phosphate backbone can induce electrostatic repulsion with protein targets and sensor surfaces, leading to reduced binding affinity and stability under physiological conditions [12, 13]. The need for extensive assay optimization and limited data on long‐term biosensor stability further constrain their clinical translation. Overall, biosensors targeting SOST remain relatively scarce, with most existing strategies focused on either immunoassays or aptamer‐based systems. This underscores a persistent need for alternative detection platforms capable of the robust, sensitive, and rapid quantification of SOST across diverse clinical samples.

Recently, short synthetic peptides, particularly those identified through biopanning techniques employing M13 bacteriophages displaying random peptide libraries, have garnered increasing attention as alternative biorecognition elements. Biopanning enables the selection of peptides with high specificity and affinity toward a given target, facilitating the development of tailored recognition motifs [14, 15]. Due to their intrinsic stability, facile synthesis, and tunable binding characteristics [16], such peptide receptors offer a robust and cost‐effective platform for SOST detection. Aptamers, while highly programmable, often face instability in complex biological matrices, whereas antibodies, despite their well‐established specificity, are constrained by long production times and high costs. By integrating the complementary advantages of both, peptides provide a stable, tunable, and cost‐effective platform for reliable biomolecular recognition in challenging biological environments [17, 18]. On this basis, peptides offer a compelling alternative to conventional biorecognition elements.

Building on the molecular recognition of SOST by these specific receptors, the subsequent challenge lies in selecting a detection strategy capable of fully leveraging the biological specificity of peptides. Electrochemical biosensing has emerged as a particularly powerful approach in this context, owing to its inherent sensitivity, rapid response times, and compatibility with miniaturized, cost‐effective devices [19]. Unlike conventional immunoassays or imaging‐based techniques, electrochemical methods enable the direct transduction of biochemical interactions into quantifiable electrical signals, often with minimal sample preparation [20, 21]. Moreover, their adaptability to point‐of‐care settings [22] and potential for large‐scale clinical implementation [23] make them ideally suited to meet the pressing demand for reliable SOST detection.

Hydrogels have recently gained prominence as platforms in biosensing due to their high biocompatibility and tunable physicochemical properties [24]. However, few functional hydrogels simultaneously offer both excellent antifouling capabilities and enhanced electrical conductivity—features essential for dependable electrochemical detection [25]. Derived from chitin by deacetylation, chitosan (CS) is a potent amino polysaccharide consisting of glucosamine as its monomeric unit, offering abundant primary amine groups. CS contributes notable biocompatibility, film‐forming capability, and an abundance of amine groups, which facilitate receptor immobilization and improve the structural stability of the hydrogel matrix [26, 27]. In parallel, the zwitterionic (ZI) polymer carboxybetaine methacrylate (CBMA) forms a strong hydration layer on the hydrogel surface, effectively preventing nonspecific protein adsorption while maintaining high biocompatibility [28]. In several representative hydrogel‐based biosensing studies, polymeric matrices combined with antibodies or aptamers have been explored to enable molecular recognition and signal transduction. While these systems demonstrate high target specificity, prior studies have discussed challenges associated with maintaining antifouling performance and sensing robustness in complex biological environments, as well as limitations in electrochemical signal transduction when nonconductive hydrogel networks are employed [29, 30]. These challenges highlight the need for multifunctional hydrogel architectures that can simultaneously support antifouling performance, stable bioreceptor presentation, and efficient electrochemical communication.

**FIGURE 1:**
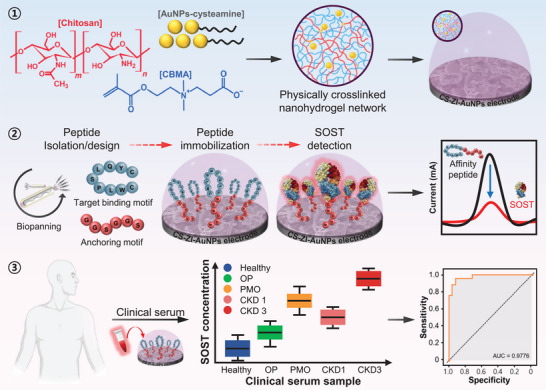
Schematic illustration of the CS–ZI–AuNPs nanohydrogel‐based electrochemical biosensor for SOST detection. Abbreviations: CBMA, carboxybetaine methacrylate; AuNPs, gold nanoparticles; SOST, sclerostin; OP, osteoporosis; PMO, postmenopausal osteoporosis; CKD, chronic kidney disease.

Herein, we employ a chitosan–zwitterionic–gold nanoparticle (CS–ZI–AuNPs) nanohydrogel as a multifunctional matrix for the electrochemical peptide‐based sensing of SOST (Figure 1). By integrating the complementary properties of CS, zwitterionic components, and AuNPs, the nanohydrogel forms a robust and versatile interface that preserves high biological fidelity in complex sample environments. In contrast to previously reported dual‐component hydrogel systems, in which antifouling performance and electrochemical transduction are typically addressed separately, the CS‐ZI‐AuNPs nanohydrogel integrates structural support, antifouling functionality, and electroactive components within a single unified interface.

This multifunctional platform significantly enhances the electrochemical transduction of peptide–SOST interactions, leading to improved sensitivity and reproducibility. The combined antifouling performance, receptor accessibility, and electrochemical responsiveness establish this nanohydrogel as a highly effective foundation for the development of reliable, high‐performance SOST biosensors.

## 2 Results and Discussion

### 2.1 Identification and Redesigning of Affinity Peptide for SOST Detection

To evaluate the specific binding efficiency of M13 phages toward SOST, biopanning was performed using a random peptide library (Ph.D.‐C7C library). The yield progressively increased through four rounds of biopanning, indicating successful enrichment of peptides with high affinity for SOST (Figure S1A). The amino acid sequences identified from the enriched phages are listed in Table S1. Subsequently, several ELISAs were conducted to determine the candidate with the highest binding affinity among the four selected clones. The 4R‐36 phage exhibited the strongest binding to SOST with minimal non‐specific interactions, as confirmed by the optical density (OD) values in the ELISA (Figure S1B). We further assessed the binding ability of 4R‐36 phages to SOST across a range of phage concentrations. As shown in Figure S1C, the binding affinity increased with increasing phage concentrations from 10^6^ to 10^12^ PFU/mL. Finally, the binding affinity of 4R‐36 phages was evaluated at varying SOST concentrations, revealing a concentration‐dependent increase in binding affinity (Figure S1D). In short, the results shown in Figure S1 demonstrate the successful identification of peptides with high specific affinity for SOST.

### 2.2 Physicochemical Properties of the CS–ZI–AuNPs Nanohydrogel

A series of physicochemical characterizations was conducted to confirm the formation of the CS–ZI–AuNPs nanohydrogel. The chemical composition and bonding states of the hydrogel were elucidated using Raman spectroscopy, attenuated total reflectance Fourier‐transform infrared (ATR‐FTIR) spectroscopy, and X‐ray photoelectron spectroscopy (XPS).

**FIGURE 2:**
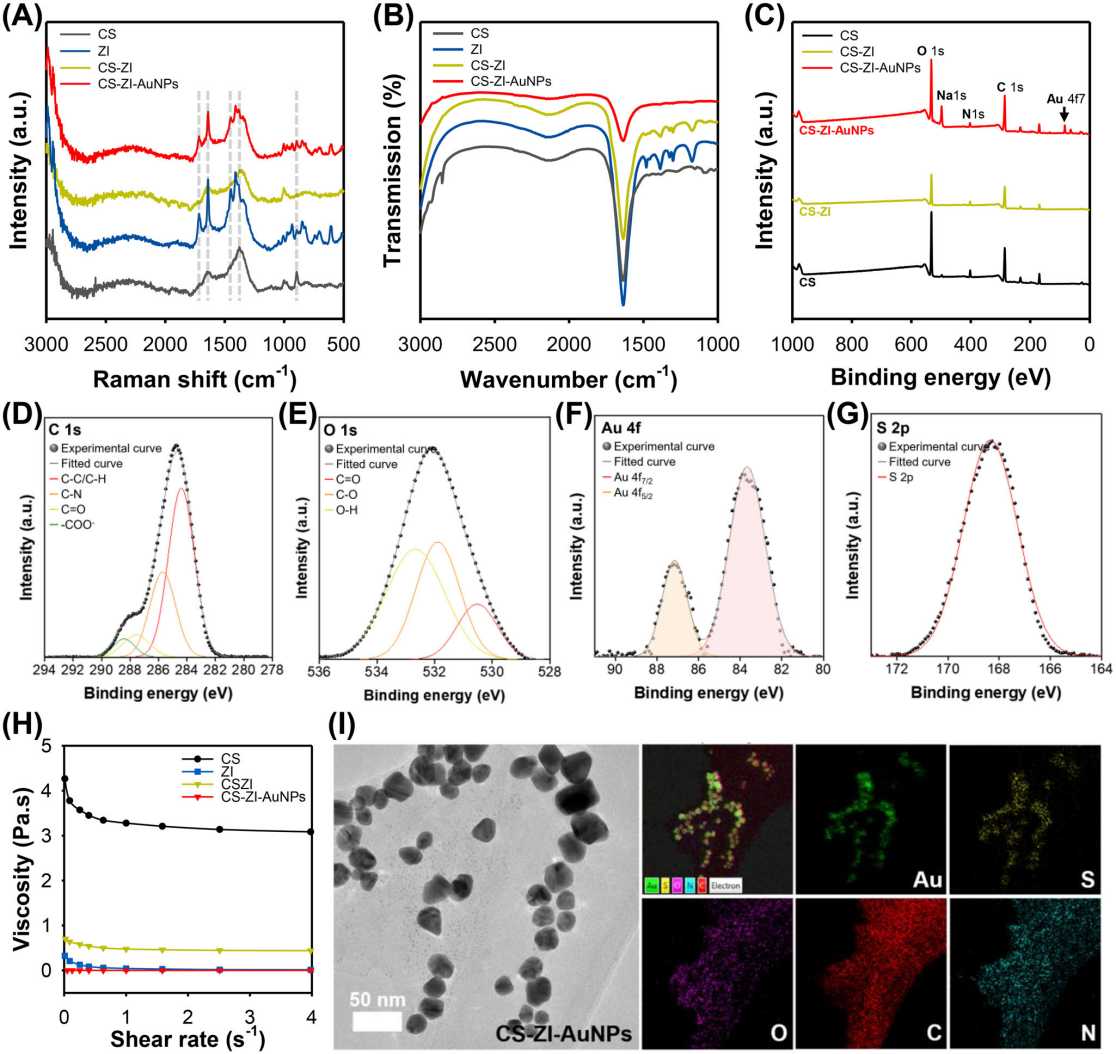
Physicochemical characterization of the CS–ZI–AuNPs nanohydrogel. (A) Raman and (B) ATR‐FTIR spectra of CS, ZI, CS–ZI, and CS–ZI–AuNPs. (C) XPS survey spectra of CS, CS–ZI, and CS–ZI–AuNPs. (D–G) High‐resolution XPS spectra of the CS–ZI–AuNPs nanohydrogel: (D) C 1s, (E) O 1s, (F) Au 4f, and (G) S 2p. (H) Rheological flow sweep profiles of CS, ZI, CS–ZI, and CS–ZI–AuNPs. (I) FE‐TEM image and EDS elemental mapping of the CS–ZI–AuNPs nanohydrogel.

As shown in Figure 2A, the Raman spectrum of CS displays a pronounced band at ≈1650 cm^−1^, corresponding to the Amide I vibration (C═O stretching), which is closely associated with the degree of acetylation. The peak at ≈1380 cm^−1^ is attributed to CH_3_ bending vibrations, while the characteristic band at ≈890 cm^−1^ arises from β‐(1→4) glycosidic link vibrations, serving as a distinctive marker for distinguishing CS. In contrast, CBMA monomer exhibits a prominent band at ≈1720 cm^−1^ assigned to the ester C═O stretching of the methacrylate moiety, along with a band at ≈1650 cm^−1^ corresponding to carboxylate C═O stretching, reflecting its zwitterionic character. A distinct peak at ≈1420 cm^−1^, resulting from CH_2_ bending modes in the methacrylate backbone, is also observed in the Raman spectrum of CBMA ZI. In CS–ZI, electrostatic interactions and hydrogen bonding cause the individual peaks to shift and merge, leading to the emergence of a new peak at ≈1360 cm^−1^. This shift indicates changes in the local chemical environment and confirms successful chemical interaction between the two components. For CS–ZI–AuNPs, signal enhancement and increased background noise at low wavenumbers are attributed to the surface‐enhanced Raman scattering effect induced by the AuNPs. As shown in Figure 2B, the ATR‐FTIR spectrum of CS displays characteristic absorptions at ≈1100 cm^−1^ (C─O─C/C─O stretching) and in the 1550‒1600 cm^−1^ range (N─H bending/C─N stretching). In contrast, CBMA monomer exhibits multiple bands across the 1200–1400 cm^−1^ region, attributable to CH_2_/CH_3_ bending and the C─O/C─N modes of the methacrylate backbone. Following conjugation, these modes converge into intermediate‐frequency features, consistent with altered intermolecular interactions and alterations in the local vibrational environment.

The elemental composition of the CS–ZI–AuNPs nanohydrogel was confirmed by XPS analysis. As shown in Figure 2C, the XPS survey spectrum revealed the presence of C, O, N, and Au, confirming the successful synthesis of the hydrogel. In the C 1s spectrum (Figure 2D), peaks were observed at 284, 286, 287.8, and 288.4 eV, corresponding to C─C/C─H, C─N, C═O, and ─COO^−^, respectively. The O 1s spectrum (Figure 2E) exhibited peaks at 530.5, 532, and 533.8 eV, attributed to C═O, C─O, and O─H groups, respectively. For the Au 4f spectrum (Figure 2F), the characteristic peaks at 83.9 and 87.7 eV were assigned to Au4f_7/2_ and Au4F_5/2_, respectively. In addition, the S 2p spectrum (Figure 2G) showed a peak at 168 eV, corresponding to S2p_1/2_. The presence of C 1s, O 1s, Au 4f, and S 2p peaks confirmed the incorporation of CS, ZI, and AuNPs into the hydrogel network.

Subsequently, rheological analysis was conducted to evaluate whether the CS–ZI–AuNPs nanohydrogel exhibited suitable viscosity for uniform spreading during drop‐casting onto the electrode surface. As shown in Figure 2H, CS exhibited an initial viscosity of approximately 3.5 Pa·s, which decreased progressively with increasing shear rate. Other individual components exhibited shear‐thinning behavior starting below 1 Pa·s. Notably, the final composite nanohydrogel began to show a viscosity reduction below 0.001 Pa·s, a property that facilitates uniform spreading during deposition while preventing excessive flow, thus ensuring consistent and homogeneous film formation on the electrode surface. Rheological reproducibility for CS, ZI, CS–ZI, and CS–ZI–AuNPs are presented in Figure S2.

The morphology of the CS–ZI–AuNPs nanohydrogel was further characterized using field‐emission transmission electron microscopy (FE‐TEM) and energy‐dispersive X‐ray spectroscopy (EDS) mapping. As shown in Figure 2I, the AuNPs appeared randomly distributed, while the CS–ZI hydrogel matrix surrounded the AuNPs via cysteamine linkage, as confirmed by the presence of Au, S, O, C, and N. The corresponding EDS spectra and quantitative elemental compositions (at.%) are provided in Figure S3. The extensive characterization results, as shown in Figure 1, demonstrate that the three components are successfully and seamlessly integrated into a nanocomposite hybrid hydrogel, which serves as an electrode‐immobilized material to enhance the performance of the biosensor.

### 2.3 Electrochemical Properties of the CS–ZI–AuNPs Nanohydrogel

The electrochemical properties of CS–ZI–AuNPs nanohydrogel‐modified electrodes were evaluated using differential pulse voltammetry (DPV), electrochemical impedance spectroscopy (EIS), and cyclic voltammetry (CV). As a first step, variations in current and resistance were measured for electrodes modified with each material. Figure S4A shows the DPV results, with the bare Au electrode serving as the baseline. Modification with CS increased the peak current, reflecting enhanced electron transfer kinetics due to the conductive and film‐forming properties of CS. In contrast, the CBMA ZI coating reduced the peak current, consistent with its charge‐neutralizing effect that hinders interfacial electron transfer. The CS–ZI composite produced an intermediate peak current, indicating a balance between the conductivity of CS and the charge‐screening effect of CBMA ZI. The incorporation of AuNPs significantly amplified the current, attributed to their high surface area and catalytic activity that facilitated electron transfer. This synergistic effect, that is, CS promoting charge transport, ZI reducing nonspecific interactions, and AuNPs enhancing conductivity, resulted in markedly improved electrochemical performance. As shown in Figure S4B, the EIS response reflects a balance between ionic transport and antifouling behavior at the interface. CS modification promotes ion conduction, resulting in a reduced interfacial resistance, whereas the zwitterionic CBMA layer increases resistance due to the formation of a strongly hydrated antifouling layer. Notably, the CS–ZI composite exhibits an intermediate resistance, indicating a synergistic and complementary interplay between enhanced ionic conductivity and surface hydration at the hybrid interface. The incorporation of AuNPs further lowered resistance, supporting their role in facilitating electron transfer. The CV response of electrodes modified with CS (Figure S5A), ZI (Figure S5C), CS–ZI (Figure S5E), and CS–ZI–AuNPs (Figure S5G) was recorded at scan rates from 10 to 100 mV/s, with corresponding peak currents shown in Figure S5B,D,F,H, respectively. In all cases, the peak current increased proportionally with the scan rate, confirming diffusion‐controlled redox processes. Among the four modifications, the CS–ZI–AuNPs electrode exhibited an i_pa_/i_pc_ ratio close to unity, whereas the others deviated slightly from 1, indicating that the incorporation of AuNPs imparted more reversible redox behavior and enhanced electrochemical stability. The CS–ZI–AuNPs electrochemical characterization results, shown in Figures S4 and S5, exhibited synergistic integration with enhanced electrical conductivity and biosensor performance, demonstrating their potential for advanced sensing applications.

### 2.4 Antifouling Property Evaluation

**FIGURE 3:**
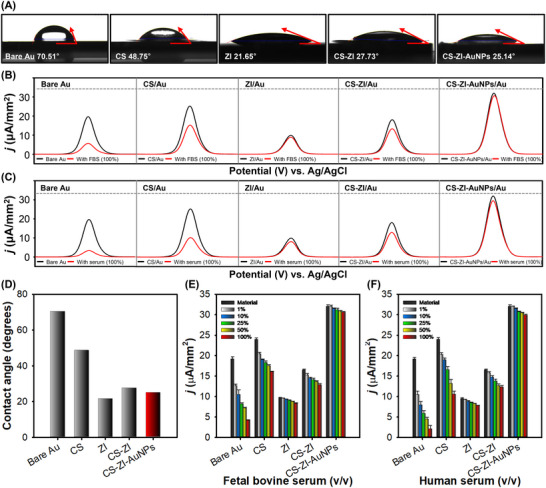
Antifouling properties of different surface modifications. (A) Water contact angles measured on bare Au, CS, ZI, CS–ZI, and CS–ZI–AuNPs. DPV responses of bare Au, CS, CS–ZI, and CS–ZI–AuNPs‐modified electrodes with (B) FBS and (C) human serum. (D) Statistical analysis of WCA measured on modified surfaces. Mean current densities of the bare Au, CS‐coated, ZI‐coated, CS–ZI‐coated, and CS–ZI–AuNPs‐coated electrodes after incubation in different concentrations of (E) FBS and (F) human serum.

Next, we investigated the antifouling ability of the CS–ZI–AuNPs nanohydrogel, as a hydrophilic surface is essential for the biosensor interface to reduce nonspecific adsorption. The hydrophilicities of the surfaces treated with each material were evaluated by water contact angle (WCA) measurements. Figure 3A shows the WCA images of bare Au treated with each material. The WCA was lowest on the ZI‐treated surface (21.65°), followed by CS–ZI–AuNPs (25.14°), CS–ZI (27.73°), and CS (48.75°). In contrast, the bare Au surface (70.51°) exhibited the highest WCA. As shown in Figure 3D, the CS–ZI–AuNPs nanohydrogel exhibited a significant reduction in the WCA, measuring less than half that of the bare Au surface. This result indicates that the CS–ZI–AuNPs nanohydrogel exhibits super‐hydrophilic properties.

To further validate the surface properties, the antifouling performance of each modification was assessed by monitoring the DPV signal response of electrodes before and after exposure to 100% fetal bovine serum (FBS) (Figure 3B) and 100% human serum (Figure 3C). As expected, human serum induced a greater signal reduction than FBS due to its higher content of impurities and diverse proteins.

Among the five coatings, bare Au and the CS‐ and CS–ZI‐modified electrodes showed a pronounced decrease in current, whereas the ZI‐ and CS–ZI–AuNPs‐modified electrodes exhibited negligible signal loss even in FBS and human serum, underscoring their superior antifouling capability and suitability for reliable biosensing in complex biological matrices. Similar zwitterionic (ZI) interfaces have been reported to effectively suppress non‐specific adsorption in human serum [31], yet such systems often suffer from fluctuations in DPV signals owing to limited electrical conductivity and mechanical stability. In contrast, by integrating CS and AuNPs within the ZI hydrogel matrix, the present platform retains robust antifouling characteristics while simultaneously enhancing charge transport and structural integrity. This synergistic design enables stable and reproducible current responses even under complex biological conditions. Subsequently, the peak current values extracted from DPV measurements were quantitatively compared across electrodes treated with varying concentrations (1%–100%) of FBS and human serum. As shown in Figure 3E, substantial current reductions were observed for bare Au and the CS‐ and CS–ZI‐modified electrodes, whereas the electrodes modified with ZI and CS–ZI–AuNPs maintained relatively stable current levels even after extended exposure to FBS, confirming their robust antifouling properties. When exposed to human serum (Figure 3F), which contains a higher abundance of proteins and impurities than FBS, a slightly greater decrease in peak currents was observed for all samples. Notably, the ZI‐ and CS–ZI–AuNPs‐modified electrodes still exhibited minimal current loss, demonstrating that the superior antifouling properties originated from the CBMA ZI, and that the final nanohydrogel CS–ZI–AuNPs achieved a level of performance comparable to that of CBMA ZI.

Building upon the electrochemical evaluation, the antifouling capability of the CS–ZI–AuNPs nanohydrogel was further investigated on CS–ZI–AuNPs‐modified gold electrodes using surface plasmon resonance (SPR) measurements (Figure S6). PBS served as the baseline, while 100% fetal bovine serum (FBS) and 100% human serum were employed to rigorously evaluate non‐specific adsorption. Both biological fluids exhibited negligible binding responses, and all interactions were completely eliminated upon washing. These results provide further evidence that the CS–ZI–AuNPs nanohydrogel effectively suppresses protein adsorption even in highly complex biological environments.

### 2.5 Analytical Performances of the CS–ZI–AuNPs Nanohydrogel Biosensor

The P3 peptide sequences identified via M13 bacteriophage‐based biopanning exhibit a defined N‐to‐C terminal orientation, in which the N‐terminus is exposed for specific target recognition while the C‐terminus remains tethered to the phage backbone. Guided by this orientation, the selected peptides were re‐synthesized with a flexible ‒GGSGGS‒ linker introduced at the C‐terminus to ensure sufficient conformational freedom and favorable presentation on the sensor surface. The detailed sequences of the synthesized peptides are summarized in Table S2.

**FIGURE 4:**
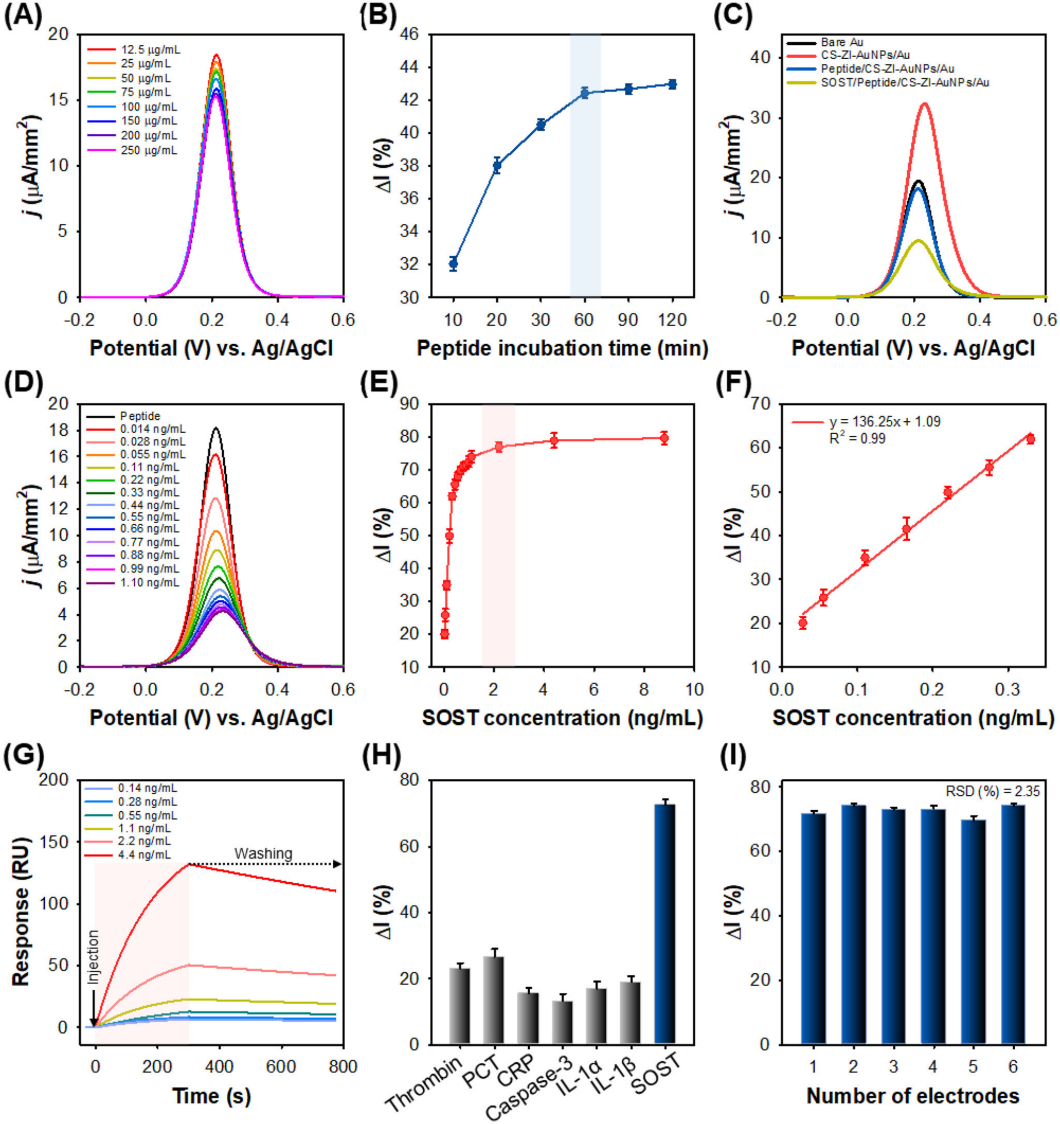
Evaluation of the developed biosensor. (A) DPV responses of peptide/CS–ZI–AuNP‐modified electrodes at different peptide concentrations. (B) Optimization of peptide incubation time for immobilization. (C) DPV signals were recorded at each fabrication step. (D) DPV responses over a wide SOST concentration range (0.014–1.1 ng/mL); a fresh electrode was used for each concentration; the curve shows the mean. (E) Calibration plot of Δ*I* vs. SOST concentration. (F) Linear dynamic range as a function of SOST concentration. (G) SPR sensogram for peptide receptor–SOST binding kinetics. (H) Specificity analysis. (I) Reproducibility test. All measurements were performed in triplicate, and error bars indicate the standard deviation.

Subsequently, EDC/NHS‐mediated coupling chemistry was employed to activate the C‐terminal carboxyl groups of the peptides, enabling their covalent conjugation to the amine groups of chitosan (CS) within the CS–ZI–AuNPs nanohydrogel. This conjugation strategy effectively anchors the peptides through the C‐terminus while maintaining the N‐terminus accessible for efficient target binding. Finally, the CS–ZI–AuNPs nanohydrogel‐modified electrodes were functionalized with the synthesized peptides at varying concentrations using the same EDC/NHS chemistry, as illustrated in Figure 4A. A gradual decrease in current density with increasing peptide concentration (12.5–200 µg/mL) confirmed stable and efficient peptide immobilization on the CS–ZI–AuNPs‐modified electrode. The immobilization time was assessed by monitoring the relative current change (Δ*I*) before and after peptide immobilization at various time points (Figure 4B). Δ*I* was calculated using Equation 1[32]:

(1) $$\Delta I\;\left(\%\right)=\left(I_{0}-I\right)/I_{0}\times 100$$

where *I*_0_ and *I* are the oxidation peak current densities of the CS–ZI–AuNPs‐modified electrode before and after peptide immobilization, respectively.

The construction of the biosensor was validated by DPV measurements (Figure 4C). Compared with bare Au, electrodes modified with CS–ZI–AuNPs exhibited markedly enhanced DPV responses due to the high conductivity of the nanohydrogel. Subsequent immobilization of the peptide at optimized concentration and incubation time led to a pronounced decrease in current density, and final binding of the target to the peptide caused a near‐complete reduction of the peak current. These results confirmed successful biosensor fabrication and demonstrated its capability for target detection. Under optimized conditions, the electrochemical response of the biosensor was evaluated across a SOST concentration range of 0.014–1.1 ng/mL (Figure 4D). As the SOST concentration increased, the DPV response progressively decreased. Figure 4E shows that Δ*I* increased in a concentration‐dependent manner and reached saturation at 2.2 ng/mL. As shown in Figure 4F, the DPV response had a linear relationship with SOST concentration over the range of 0.0275–0.33 ng/mL (*y* = 136.25*x* + 18.37, *R* ^2^ = 0.99), indicating reliable quantitative performance. Here, *y* and *x* represent Δ*I* (%) and SOST concentration (ng/mL), respectively.

SPR analysis was performed to further investigate the binding kinetics of SOST to the peptide receptor, which was immobilized on CS–ZI–AuNPs‐treated gold electrodes following the same protocol as in the electrochemical measurements. While the SPR response was less sensitive than the electrochemical measurements (Figure 4G), a concentration‐dependent increase in the response was observed over the range of 0.14–4.4 ng/mL, with minimal change in response at the lowest SOST concentration (0.14 ng/mL). The kinetic fitting yielded an association rate constant (*k*_on_) of 1.3 × 10^5^ M^−1^·s^−1^ and dissociation rate constant (*k*_off_) of 2.4 × 10^−14^ s^−1^, corresponding to a limit of detection (LOD) of 39.6 ng/mL. These results confirm the formation of strong and specific peptide–SOST interactions, while highlighting that the electrochemical (EC) biosensor provides markedly higher sensitivity, achieving a LOD of 26.4 pg/mL. These findings indicate that the biopanning‐derived peptide enables highly sensitive target recognition on the sensor surface, supporting the effectiveness of the developed electrochemical platform.

A specificity test was performed against multiple disease biomarkers, including thrombin, procalcitonin (PCT), C‐reactive protein (CRP), caspase‐3, interleukin‐1α (IL‐1α), and interleukin‐1β (IL‐1β), to verify biosensor selectivity and assess potential nonspecific binding (Figure 4H). The Δ*I* values for these proteins were negligible compared with those of SOST, confirming excellent selectivity. To assess temporal stability, peptide‐functionalized electrodes were stored at 4°C and monitored over 18 d (Figure S7). The biosensor maintained excellent stability, showing only a slight decrease in current density with a signal loss of approximately 9.39%. Reproducibility was also examined (Figure 4I) using six peptide–CS–ZI–AuNPs‐modified electrodes incubated with SOST for 0.5 h, yielding a relative standard deviation (RSD) of 2.35%, confirming strong reproducibility.

### 2.6 Correlation Assay of the CS–ZI–AuNPs Biosensor with a Commercially Available ELISA Kit

**FIGURE 5:**
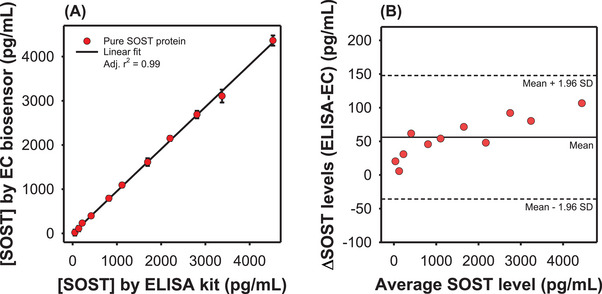
Comparison of the developed EC biosensor and a commercial ELISA kit for pure SOST protein detection. (A) Correlation analysis between EC biosensor and ELISA measurements. (B) Bland–Altman plot comparing EC biosensor and ELISA results.

Before application to patient samples, the analytical performance of the EC biosensor was benchmarked against a commercial ELISA kit using purified SOST across the ELISA‐specified concentration range (0–4400 pg/mL). Eleven discrete concentrations were tested in parallel, producing a near‐perfect correlation with the ELISA (*R* ^2^ = 0.99, Figure 5A). Bland–Altman analysis further confirmed concordance, with all measurements falling within the mean ± 1.96 standard deviation (SD), indicating negligible systematic bias (Figure 5B). Collectively, these results demonstrate that the EC biosensor provides accurate and reproducible quantification across clinically relevant concentrations, establishing a strong foundation for subsequent validation in patient‐derived samples.

### 2.7 SOST Detection in the Human Patient's Serum

**FIGURE 6:**
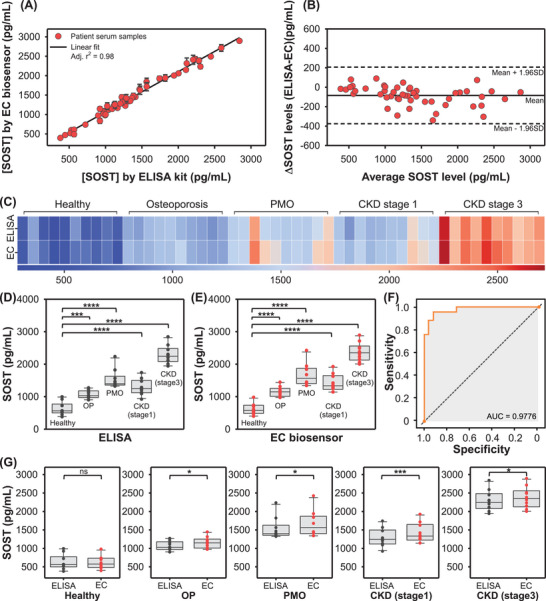
Quantification of SOST in 50 patient serum samples using the EC biosensor and a commercial ELISA kit. (A) Correlation analysis between EC biosensor and ELISA measurements. (B) Bland–Altman plot comparing EC biosensor and ELISA results. (C) Heatmap representation of SOST concentrations quantified by both methods. Color gradients correspond to increasing signal values, illustrating higher concentrations in the disease groups. Box plots of SOST concentrations measured with (D) the ELISA kit and (E) the EC biosensor. (F) ROC curve for the EC biosensor vs. the ELISA kit reference. (G) Within‐group comparison between the ELISA kit and EC biosensor across the clinical groups. Significance levels from paired t‐tests are indicated (ns, not significant; ^*^ *p* < 0.05, ^**^ *p* < 0.01, ^***^ *p* < 0.001, ^****^ *p* < 0.0001). Abbreviations: OP, osteoporosis; PMO, postmenopausal osteoporosis; CKD, chronic kidney disease. All experiments were performed in triplicate.

Finally, to validate the clinical applicability of the CS–ZI–AuNPs nanohydrogel biosensor, SOST levels were quantified in patient serum samples across five groups, including healthy controls, and directly compared with measurements from a commercial ELISA kit (Figure 6). Serum samples from 50 patients, 10 each from the following groups: healthy control, OP, postmenopausal osteoporosis (PMO), and CKD stages 1 and 3, were analyzed using both methods. Correlation analysis demonstrated excellent agreement, with SOST concentrations measured in the 50 clinical samples showing a nearly perfect linear relationship with ELISA results (*R* ^2^ = 0.98) (Figure 6A). Complementary Bland–Altman analysis confirmed this consistency, as all samples fell within the mean ± 1.96 SD, underscoring the reliability of the EC biosensor for clinical SOST quantification (Figure 6B). A heatmap further illustrates the strong concordance between the two methods (Figure 6C), while the quantitative values for all samples are provided in Table S3.

Group‐wise serum SOST concentrations quantified by ELISA and the electrochemical (EC) biosensor are presented as box plots in Figure 6D,E, respectively. Consistent with clinical expectations, healthy controls exhibited the lowest SOST levels, whereas progressively elevated concentrations were observed across the OP, CKD stage 1, PMO, and CKD stage 3 cohorts. Previous studies have reported serum SOST levels below approximately 800 pg/mL (36 pmol/L) in healthy individuals, with increases up to ≈2112 pg/mL (96 pmol/L) in OP patients. In the present cohort, the measured values fell well within these established ranges, with OP and PMO samples approaching ≈2000 pg/mL, indicating that the EC biosensor accurately captures physiologically relevant variations in circulating SOST. Notably, CKD stage 3 patients displayed markedly elevated SOST concentrations approaching ≈3000 pg/mL, in agreement with prior reports linking renal dysfunction to increased circulating SOST levels. To quantitatively assess agreement between the two analytical modalities, receiver operating characteristic (ROC) analysis was performed by comparing EC biosensor outputs with ELISA measurements (Figure 6F). The resulting area under the curve (AUC) of 0.9776 demonstrates excellent concordance and strong discriminatory performance of the EC biosensor relative to the clinical reference method.

Importantly, SOST concentrations determined by the EC biosensor closely matched those obtained by ELISA, underscoring the analytical robustness and clinical relevance of the proposed platform. To further evaluate measurement consistency at the cohort level, within‐group comparisons were conducted (Figure 6G). Across all groups, EC biosensor and ELISA results were highly comparable, with no significant difference observed in healthy controls and only minor but statistically significant differences detected in the OP, PMO, and CKD stage 3 groups (*p* < 0.05), as well as in the CKD stage 1 group (*p* < 0.001). Collectively, these findings demonstrate that the EC biosensor reliably reproduces clinically meaningful SOST profiles across diverse patient populations, highlighting its strong potential for robust and translational diagnostic applications.

Based on these observations, the developed EC biosensor not only reliably quantifies SOST in comparison with conventional ELISA but also captures disease‐ and group‐specific variations, providing a clinically relevant platform for monitoring bone and renal health. While this pilot study demonstrates meaningful and clinically relevant results, future studies with larger, demographically diverse cohorts and multiple comparators will further refine and validate the diagnostic potential of the EC biosensor. The clinical characteristics of all 50 patients included in this study are summarized in Table S4.

## 3 Conclusions

We developed an EC biosensor that integrated biopanning‐derived peptides with a multifunctional CS–ZI–AuNPs nanohydrogel interface. Comprehensive physicochemical characterization confirmed the successful synthesis of CS–ZI–AuNPs, exhibiting antifouling capacity, abundant functional groups for stable peptide immobilization, and efficient electrochemical transduction. This synergistic design enables highly sensitive, reproducible, and label‐free SOST detection in complex biological matrices. The biosensor demonstrated accurate quantification across clinically relevant concentrations, showed strong agreement with ELISA results, and achieved statistically significant discrimination between healthy individuals and patient groups, including OP, PMO, and CKD stages 1 and 3. These findings underscore the clinical relevance of the developed platform and highlight its potential for real‐time biomarker monitoring.

Given the established involvement of sclerostin (SOST) in a broad range of pathological conditions, including bone‐ and kidney‐related disorders, the present platform may be readily extended toward multiplexed biomarker detection. This could be achieved by spatially patterning distinct peptide‐based bioreceptors or by integrating multiple electrode elements within a single hydrogel matrix, thereby enabling the simultaneous and quantitative analysis of multiple disease‐relevant biomarkers. Furthermore, the CS–ZI–AuNPs nanohydrogel is fabricated through simple chemical and physical crosslinking processes, avoiding the need for complex photopolymerization steps. This streamlined fabrication strategy supports scalability, batch‐to‐batch reproducibility, and practical manufacturability. In addition, the electrode‐compatible nature of the hydrogel allows seamless integration into established diagnostic formats, including electrode‐based assay platforms and miniaturized electrochemical devices suitable for routine clinical deployment.

Collectively, these attributes underscore the strong potential of the proposed platform for multiplexed biomarker profiling, high‐throughput clinical evaluation, and broader translational diagnostic applications. Overall, this work establishes a versatile paradigm for protein biomarker diagnostics through the strategic integration of peptide bioreceptors and functional hydrogel engineering, providing a foundation for broader applications in early disease detection, multiplexed biosensing, and precision healthcare.

## 4 Experimental Section

### 4.1 Ethics Statement

The study was approved by the Ethics Review Committee of the Institutional Review Board at Chung‐Ang University (approval No. 1041078‐20250331‐BR‐095). All procedures involving human participants were conducted in accordance with the ethical standards of the institutional research committee and the Declaration of Helsinki. Written informed consent was obtained from all patients prior to sample collection.

### 4.2 Chemicals and Reagents

SOST, IL‐1α, and IL‐1β were obtained from Acrobiosystems (Newark, DE, USA). Caspase‐3 and thrombin were purchased from Novus Biologicals (Centennial, CO, USA). The random peptide‐displayed phage library (Ph.D.‐C7C library) and *Escherichia coli* (*E. coli*) strain ER2738 for biopanning were obtained from New England Biolabs (Ipswich, MA). Chitosan oligosaccharide lactate (average *M*_n_: 5 kDa, >90% deacetylation), EDC, NHS, FBS, and human serum were purchased from Sigma–Aldrich (St. Louis, MO). The human SOST ELISA kit and dimethyl sulfoxide (DMSO; extra dry) were purchased from Thermo Fisher Scientific (Waltham, MA). CBMA (3‐[[2‐(methacryloyloxy)ethyl]dimethylammonio]propionate) was purchased from TCI (Tokyo, Japan). All solutions were prepared using ultrapure water (Milli‐Q, Millipore, Bedford, MA).

### 4.3 Instrumentation

All physicochemical characterizations were performed using standard high‐resolution analytical instruments. XPS was conducted using a K‐Alpha+ system (Thermo Fisher Scientific) to analyze surface elemental composition and chemical states. High‐resolution imaging and structural analysis were carried out using FE‐TEM (JEM‐F200, JEOL). FTIR spectra were recorded using a TENSOR 27 spectrometer (Bruker) in ATR mode, and Raman spectra were obtained with a DXR2xi Raman microscope (Thermo Fisher Scientific). WCA measurements were obtained using a DSA100 drop shape analyzer (Kruss) to evaluate changes in surface wettability. Electrochemical analyses, including CV, DPV, and EIS, were performed using a CHI660 workstation (CH Instruments). SPR measurements were carried out using a Reichert SR7500DC system to monitor real‐time biomolecular interactions.

### 4.4 Biopanning Process and DNA Sequencing

Biopanning was performed according to our previous report, with minor modifications [33]. In the first round, biotinylated SOST was incubated with 1 µL of the Ph.D.‐C7C M13 phage library (1 × 10^11^ PFU/mL) under gentle shaking at 150 rpm for 1 h at room temperature. The resulting complex was transferred to streptavidin‐coated 96‐well plates (100 µL/well) and allowed to react for 10 min at 150 rpm to promote the biotin–streptavidin interaction. Excess binding sites were blocked with 0.1 mm biotin, followed by washing with PBST (0.1% Tween‐20 in PBS, pH 7.4). Bound phages were eluted with 0.2 m glycine‐HCl (pH 2.2, 5 mg/mL BSA) and immediately neutralized with Tris‐HCl (pH 9.1). The recovered phages were then amplified in *E. coli* ER2738 and quantified for subsequent rounds. From the second round onward, amplified phages were subjected to negative selection against streptavidin‐coated plates, while the PBST washing stringency was gradually increased (0.1%–0.5% Tween‐20). This process was repeated for four rounds. Enriched phages were sequenced (Solgent, Korea), and recovery yields were calculated (Table S1). Four candidate clones were ultimately obtained.

### 4.5 Affinity Peptides specific SOST

A functionalized SOST affinity peptide derived from the C7C‐mer sequence (CWLKSLQYC) was identified through biopanning and synthesized with the modified sequence (CWLPSLQYCGGSGGS) by AnyGen (>95% purity, Gwangju, Korea). The fourth residue, a lysine (K), containing an amine side chain, was replaced with a proline (P) to prevent undesired side reactions during EDC/NHS coupling. A GGSGGS linker was introduced to enhance structural flexibility. The synthesized peptides were purified by high‐performance liquid chromatography (>95% purity), characterized by liquid chromatography–mass spectrometry, lyophilized, dissolved in DMSO, and stored at −20°C until use.

### 4.6 Fabrication of CS–ZI–AuNPs Nanohydrogel

To fabricate the CS–ZI–AuNP nanohydrogel, CS (5%, w/v) was mixed with a ZI monomer solution (10% w/v) and stirred at room temperature for 6 h. In parallel, citrate‐stabilized AuNPs (20 nm) were functionalized with cysteamine by adding cysteamine (final concentration 1 mM) and stirring at room temperature for 2 h, followed by two centrifugation–redispersion cycles (12,000 *g*, 10 min) to remove excess ligand. The cysteamine‐functionalized AuNPs were subsequently added to the CS–ZI mixture, and the suspension was stirred for an additional 12 h at room temperature, yielding a homogeneous nanohydrogel.

### 4.7 Construction of the EC Biosensor Using CS–ZI–AuNPs Nanohydrogel and Affinity Peptide

Au electrodes were fabricated on a Si wafer by electron beam lithography at the National NanoFab Center (Daejeon, Korea) following established procedures. The electrodes were sequentially washed with ethanol and distilled water, immersed in piranha solution (H_2_SO_4_:H_2_O_2_ = 7:3) for 7 min, rinsed with distilled water, and dried under a N_2_ gas flow to ensure surface cleanliness. Subsequently, 15 µL of the CS–ZI–AuNP nanohydrogel was drop‐cast onto the cleaned Au electrodes and dried at 40°C for 2 h. SOST affinity peptide was conjugated to the CS–ZI–AuNP‐coated gold electrodes through EDC/NHS coupling chemistry. For activation, 200 mm EDC and 50 mm NHS were added to a 50 µg/mL solution of affinity peptide in 20 mm MES buffer (pH 6) and pre‐reacted for 30 min at room temperature with gentle shaking, converting the C‐terminal carboxylate group to NHS ester intermediates. The pre‐activated peptide solution was then incubated on the hydrogel‐modified electrodes for 1 h under gentle shaking, yielding stable amide bonds with CS's primary amines by acyl substitution reaction. Finally, recombinant SOST protein at various concentrations was incubated on the affinity peptide‐functionalized electrodes under the same conditions, and the electrodes were subsequently used for electrochemical detection.

### 4.8 Clinical Sample Preparation

Serum samples from patients with unspecified OP, PMO, and healthy controls were obtained from the Chungbuk National University Biobank, while samples from patients with CKD stages 1 and 3 were obtained from the Seoul National University Biobank. To preserve biological activity, serum samples were diluted 100‐fold with 0.1 m PBS and used for both electrochemical measurements and ELISA analysis.

### 4.9 Correlation Assay Using Commercial ELISA Kit and Developed Biosensor

Comparative analyses were conducted using both the standard SOST protein provided in the ELISA kit and the recombinant SOST protein prepared in this study to evaluate the detection performance of the developed electrochemical biosensor against the commercial ELISA kit. The proteins were serially diluted within the concentration range specified by the ELISA kit. Correlation coefficients were calculated, and Bland–Altman plots were constructed based on the calibration curves to assess concordance between the two detection methods. The detection performance using patient serum samples was evaluated following the same methodology described above.

## Conflicts of Interest

The authors declare no conflicts of interest.

## Supporting information




**Supporting File**: advs74575‐sup‐0001‐SuppMat.docx.

## Data Availability

The data that support the findings of this study are available from the corresponding author upon reasonable request.
