# Supplementary material for: Peptide Receptor‐Functionalized AuNP‐Embedded Zwitterionic Biopolymeric Nanohydrogel for Electrochemical Sclerostin Sensing
Source: Adv Sci (Weinh). 2026 Feb 25;13(26):e74575. doi: 10.1002/advs.74575 (PMC13159139; doi:10.1002/advs.74575)
Supplement: Supplementary file 1 — Supporting File: advs74575‐sup‐0001‐SuppMat.docx. [file ADVS-13-e74575-s001.docx]

Supporting Information:

Peptide Receptor-Functionalized AuNP-Embedded Zwitterionic Biopolymeric Nanohydrogel for Electrochemical Sclerostin Sensing

Hyo Jeong Yang^¶^, Jae Hwan Shin^¶^, Mingi Jo, Changyeon Lee, Hyunmin Yi, Al-Montaser Bellah H. Ali and Jong Pil Park*

H.J. Yang, J.H. Shin, Prof. J.P. Park

Department of Food Science and Technology, and GreenTech-Based Food Safety Research Group, BK21 Four, Chung-Ang University, Anseong 17546, Republic of Korea
E-mail: jppark@cau.ac.kr

M. Jo, Prof. C. Lee

Department of Chemical Engineering, Chung-Ang University, Seoul 06974, Republic of Korea

Prof. H. Yi

Department of Chemical and Biological Engineering, Tufts University, Medford, Massachusetts 02155, United States

Prof. A.M.B.H. Ali

Department of Pharmaceutical Analytical Chemistry, Faculty of Pharmacy, Assiut University, Assiut, Egypt

^¶^ These authors contributed equally to this work.

**List of Table Captions**

**Table S1**

Amino acid sequences selected during biopanning of the C7C-mer peptide library

**Table S2**

Characteristics of synthetic peptides used in this study.

**Table S3**

Comparison of SOST concentrations in patient samples measured using the developed biosensor and a commercial ELISA kit.

**Table S4**

Clinical information of the 50 patients included in this study.

**List of Figure Captions**

**Figure S1**

Characterization of SOST-binding phage clones by ELISA. (A) Recovery yield (%) of SOST-binding sequences over four rounds of biopanning. (B) Selection of SOST-specific binding phages. (C) Effect of phage concentration. (D) Effect of protein concentration. All measurements were performed in triplicate, and error bars represent standard deviations.

**Figure S2**

Reproducibility of rheology flow sweep measurements. (A) CS. (B) ZI. (C) CS-ZI. (D) CS-ZI-AuNPs.

**Figure S3**

Elemental distribution (atomic %) obtained from EDS map sum spectrum.

**Figure S4**

(A) DPV responses and (B) EIS spectra of electrodes modified with CS, ZI, CS-ZI, and CS-ZI-AuNPs.

**Figure S5**

Cyclic voltammograms recorded at different scan rates for CS, ZI, CS-ZI, and CS-ZI-AuNPs (A, C, E, G) and the corresponding peak currents (B, D, F, H) derived from the voltammograms.

**Figure S6**

SPR sensogram of PBS (baseline), 100% FBS, and 100% human serum on the CS-ZI-AuNPs nanohydrogel surface, showing antifouling.

**Figure S7**

Stability test of the developed biosensor over 18 days.

**Table S1.** Amino acid sequences selected during biopanning of the C7C-mer peptide library.

| **Name** | **Amino acid sequence (N→C terminus)** | **Frequency** |
| --- | --- | --- |
| 4R-18 | CGEGEADVC  (Hydrophobic: 22.22 %, Acidic: 33.33 %, Basic: 0 %, Neutral: 44.44 %) | 3/80 |
| 4R-25 | CSSNTVPAC  (Hydrophobic: 33.33 %, Acidic: 0 %, Basic: 0 %, Neutral: 66.67 %) | 4/80 |
| 4R-36 | CWLKSLQYC  (Hydrophobic: 33.33 %, Acidic: 0 %, Basic: 11.11 %, Neutral: 55.56 %) | 26/80 |
| 4R-40 | CNNVMHRVC  (Hydrophobic: 33.33 %, Acidic: 0 %, Basic: 22.22 %, Neutral: 44.44 %) | 3/80 |

*Analysis of amino acid sequence of peptides was performed with PEPTIDE 2.0 program (<http://peptide2.com/N_peptide_hydrophobicity_hydrophilicity.php>).

**Table S2.** Information on synthesized peptides.

| **Name** | SOST affinity peptide |
| --- | --- |
| **Amino acid sequence**  **(N→C terminus)** | Ac‒CWLPSLQYCGGSGGS (1C-9C Disulfide bond)  (Hydrophobic: 26.67 %, Acidic: 0 %, Basic: 0 %, Neutral: 73.33 %) |
| **Molecular weight** | 1554.8 |
| **Purity** | 95.2 % |

*Analysis of amino acid sequence of peptides was performed with PEPTIDE 2.0 program (<http://peptide2.com/N_peptide_hydrophobicity_hydrophilicity.php>).

**Table S3.** Comparison of SOST concentrations in patient samples measured using the developed biosensor and a commercial ELISA kit.

| **n** | **Healthy** | | **OP** | | **PMO** | | **CKD (stage 1)** | | **CKD (stage 3)** | |
| --- | --- | --- | --- | --- | --- | --- | --- | --- | --- | --- |
|  | ELISA kit (pg/mL) | EC biosensor (pg/mL) | ELISA kit | EC biosensor | ELISA kit | EC biosensor | ELISA kit | EC biosensor | ELISA kit | EC biosensor |
| **#1** | 378 ± 8 | 396 ± 17 | 1036 ± 19 | 1129 ± 11 | 1367 ± 16 | 1428 ± 27 | 1266 ± 10 | 1354 ± 21 | 2841 ± 33 | 2893 ± 38 |
| **#2** | 556 ± 5 | 544 ± 6 | 969 ± 13 | 1075 ± 16 | 1412 ± 10 | 1451 ± 24 | 922 ± 10 | 1145 ± 11 | 2003 ± 21 | 2058 ± 25 |
| **#3** | 995 ± 3 | 979 ± 6 | 1165 ± 7 | 1169 ± 15 | 2234 ± 22 | 2424 ± 21 | 1285 ± 16 | 1420 ± 13 | 2447 ± 19 | 2490 ± 24 |
| **#4** | 528 ± 8 | 697 ± 18 | 1223 ± 9 | 1446 ± 18 | 1467 ± 6 | 1849 ± 16 | 1091 ± 14 | 1142 ± 15 | 2116 ± 21 | 2315 ± 16 |
| **#5** | 445 ± 2 | 470 ± 28 | 1272 ± 14 | 1224 ± 23 | 1382 ± 6 | 1477 ± 14 | 1128 ± 9 | 1251 ± 11 | 2591 ± 17 | 2713 ± 17 |
| **#6** | 517 ± 11 | 518 ± 14 | 1116 ± 26 | 1231 ± 37 | 1322 ± 15 | 1340 ± 15 | 1228 ± 13 | 1314 ± 17 | 2203 ± 15 | 2508 ± 12 |
| **#7** | 923 ± 7 | 734 ± 16 | 1023 ± 13 | 1315 ± 22 | 1362 ± 9 | 1420 ± 15 | 1203 ± 19 | 1280 ± 21 | 2285 ± 18 | 2383 ± 18 |
| **#8** | 567 ± 9 | 608 ± 14 | 974 ± 17 | 1013 ± 16 | 1340 ± 13 | 1346 ± 24 | 1741 ± 7 | 1925 ± 15 | 1941 ± 15 | 2001 ± 18 |
| **#9** | 723 ± 4 | 738 ± 26 | 899 ± 9 | 973 ± 9 | 1564 ± 27 | 1840 ± 33 | 1566 ± 11 | 1715 ± 24 | 2105 ± 20 | 2148 ± 17 |
| **#10** | 567 ± 10 | 489 ± 3 | 1002 ± 10 | 998 ± 12 | 1820 ± 31 | 1940 ± 25 | 1475 ± 15 | 1625 ± 21 | 2304 ± 24 | 2231 ± 20 |

*Abbreviations: OP, osteoporosis; PMO, postmenopausal osteoporosis; CKD, chronic kidney disease; EC, electrochemical;

**Table S4.** Clinical information of the 50 patients included in this study.

|  | | **Healthy** | **OP** | **PMO** | **CKD (stage 1)** | **CKD (stage 3)** |
| --- | --- | --- | --- | --- | --- | --- |
| **Total patient number (n)** | | 10 | 10 | 10 | 10 | 10 |
| **Gender (n)** | **Male** | 6 | 3 | 0 | 2 | 4 |
|  | **Female** | 4 | 7 | 10 | 8 | 6 |
| **eGFR (mL/min)** | | 111.31 ± 28.23 | 85.92 ± 14.64 | 80.69 ± 32.25 | 91.88 ± 15.58 | 42.72 ± 9.05 |
| **Creatinine (mg/dL)** | | 0.77 ± 0.17 | 0.65 ± 0.15 | 1.43 ± 2.08 | 0.66 ± 0.09 | 1.78 ± 0.21 |
| **Phosphate (mg/dL)** | | 3.2 ± 0.58 | 3.4 ± 0.66 | 3.66 ± 0.67 | 3.58 ± 0.72 | 3.59 ± 0.74 |
| **Calcium (mg/dL)** | | 9.58 ± 0.36 | 8.93 ± 1.08 | 8.9 ± 0.53 | 9.3 ± 0.49 | 8.56 ± 0.79 |

*Abbreviations: OP, osteoporosis; PMO, postmenopausal osteoporosis; CKD, chronic kidney disease;

**
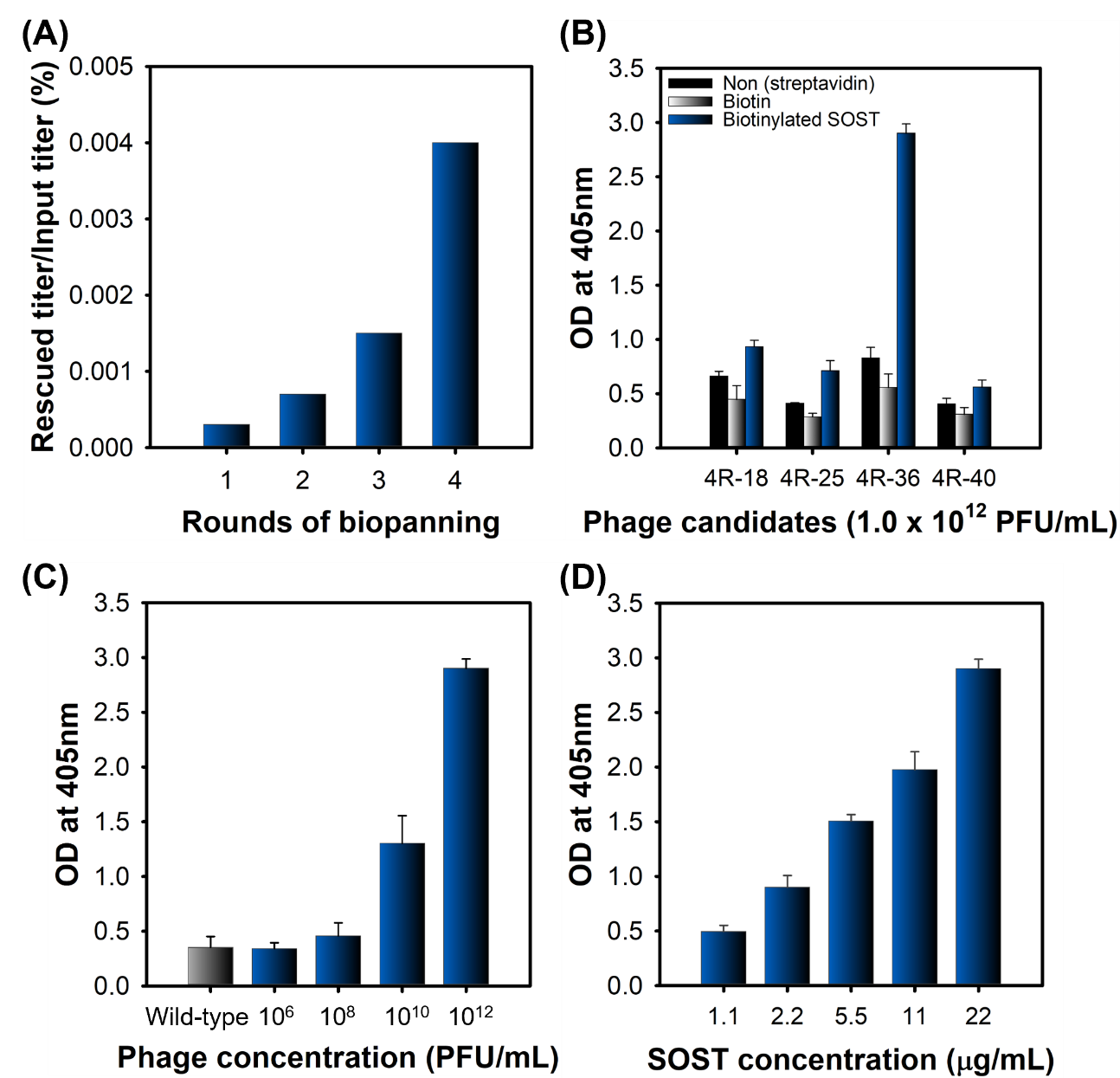
**

**Figure S1.** Characterization of SOST-binding phage clones by ELISA. (A) Recovery yield (%) of SOST-binding sequences across four rounds of biopanning. (B) Selection of SOST-specific binding phages. (C) Effect of phage concentration. (D) Effect of protein concentration. All measurements were performed in triplicate, and error bars represent standard deviations.


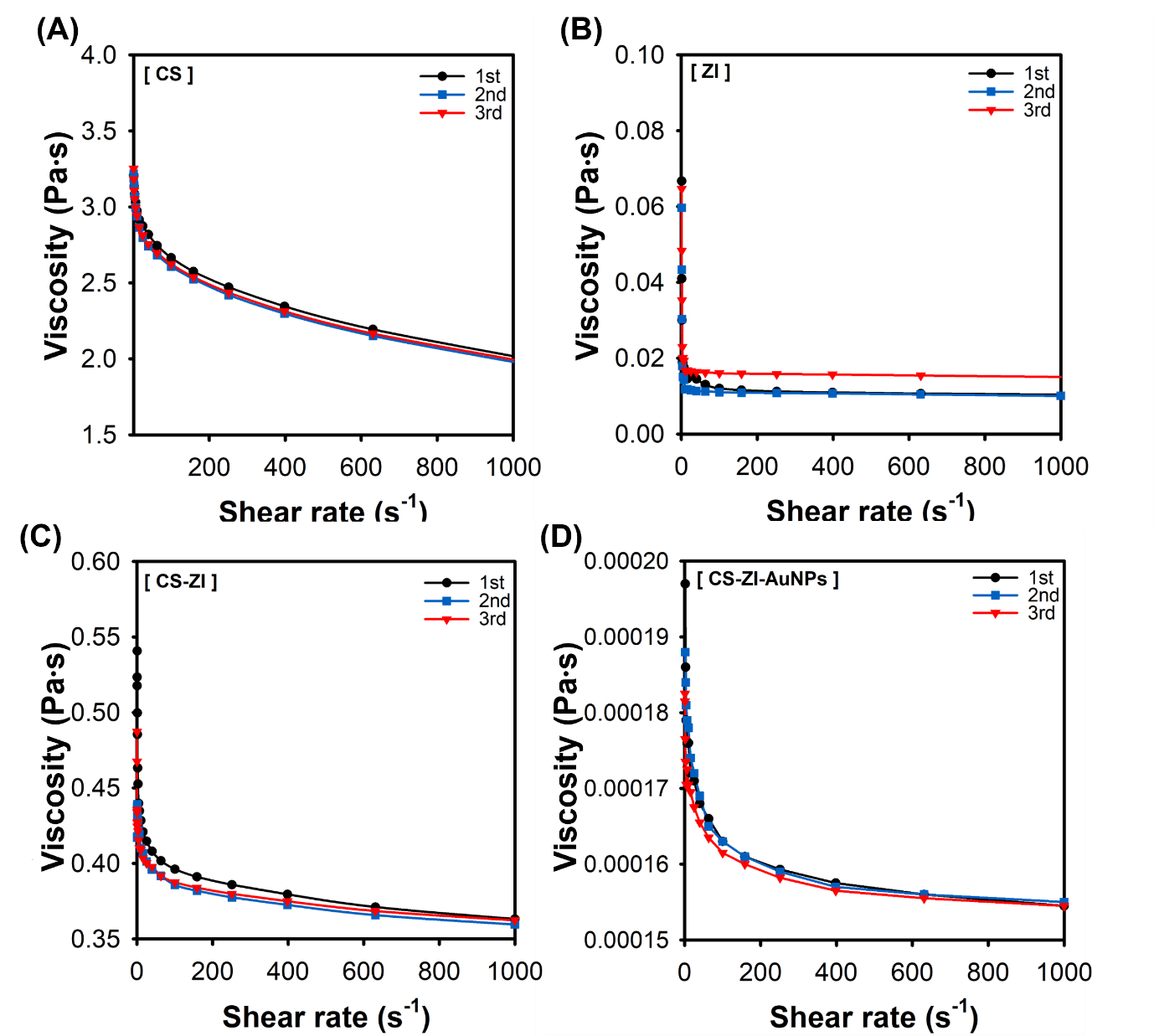


**Figure S2.** Reproducibility of rheology flow sweep. (A) CS. (B) ZI. (C) CS-ZI. (D) CS-ZI-AuNPs.


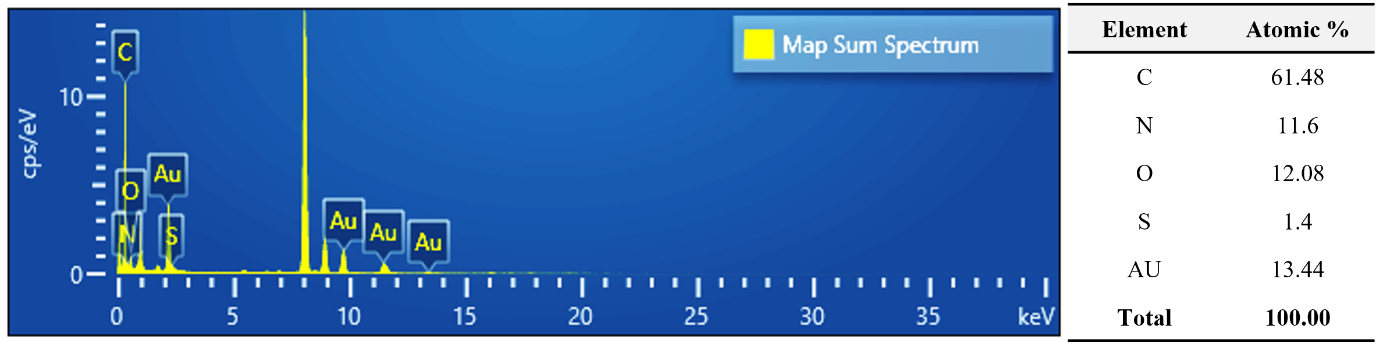


**Figure S3.** Elemental distribution (atomic %) obtained from EDS map sum spectrum.


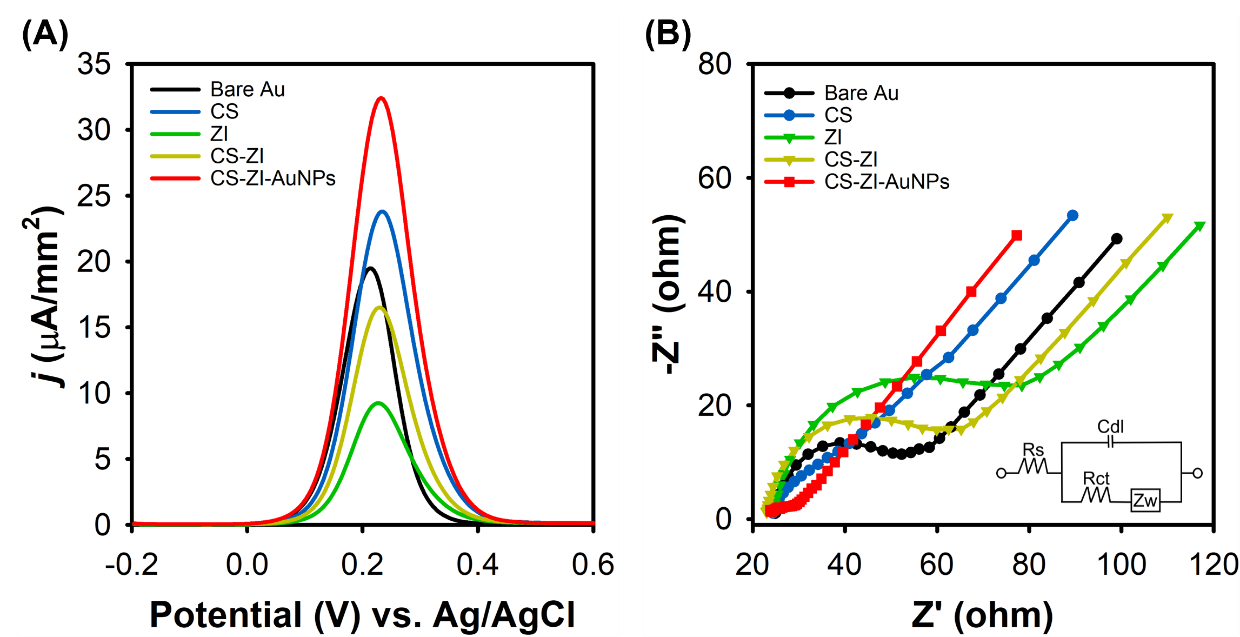


**Figure S4.** (A) DPV responses and (B) EIS spectra of electrodes modified with CS, ZI, CS-ZI, and CS-ZI-AuNPs.


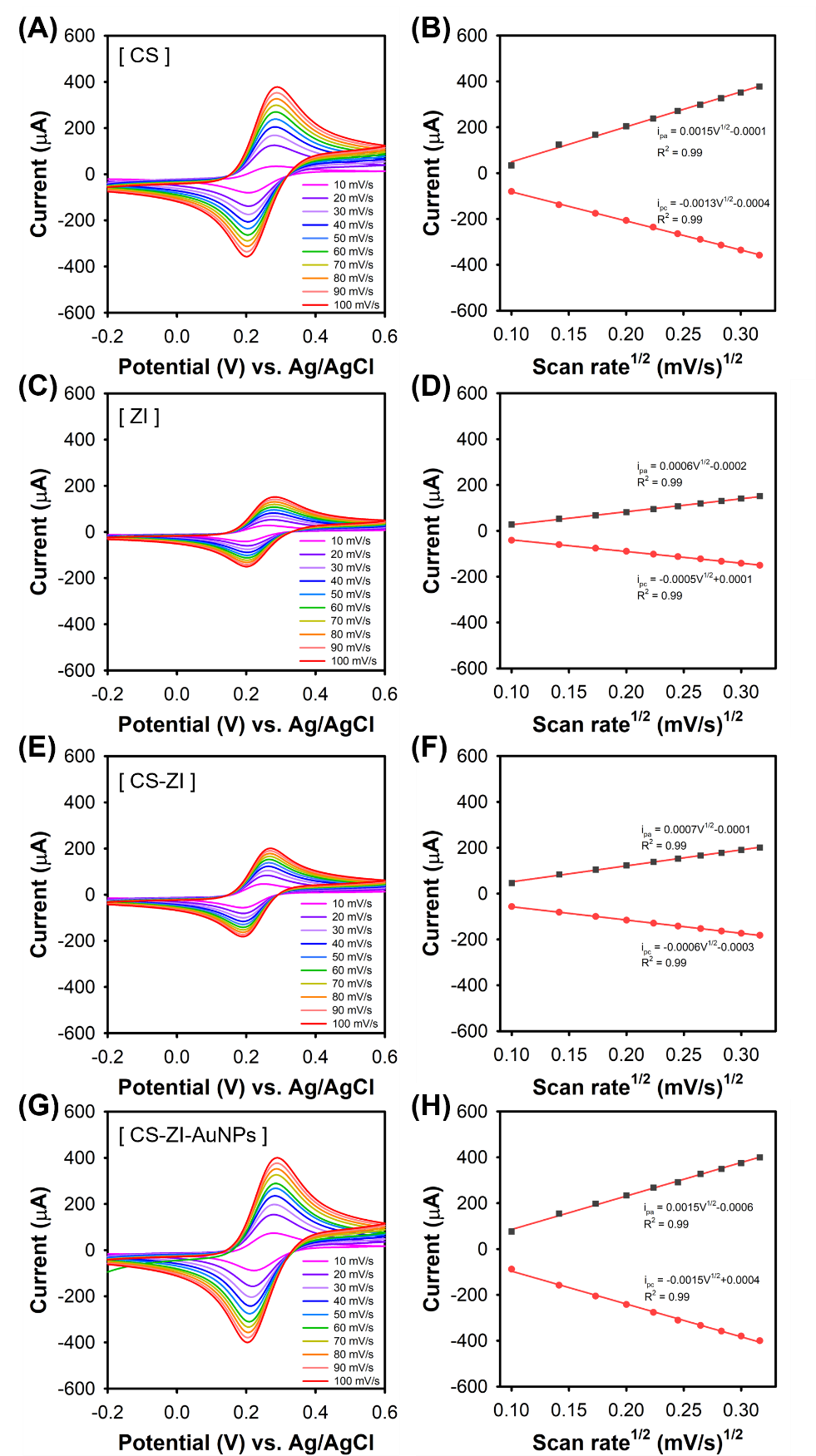


**Figure S5.** Cyclic voltammograms recorded at different scan rates for CS, ZI, CS-ZI, and CS-ZI-AuNPs (A, C, E, G), with the corresponding peak currents (B, D, F, H) derived from the respective voltammograms.


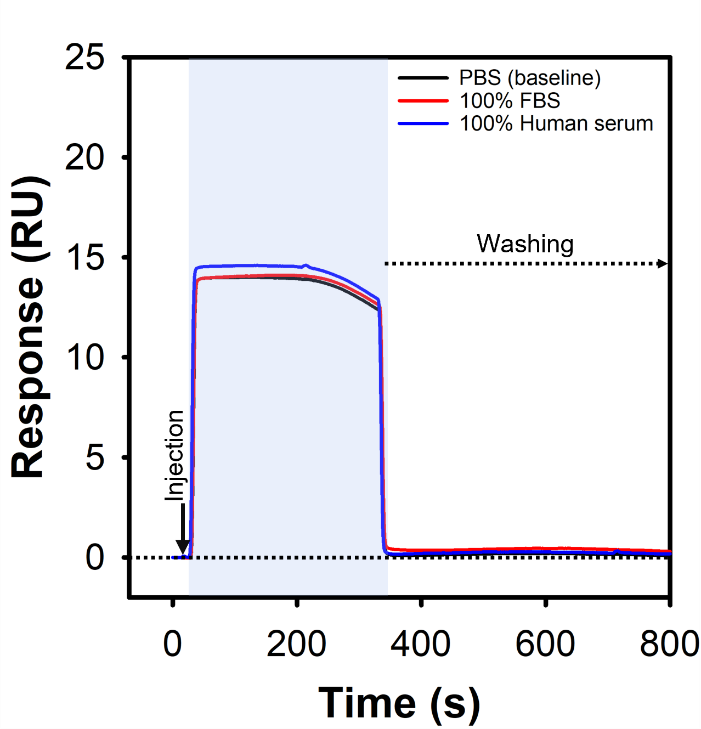


**Figure S6.** SPR sensogram of PBS (baseline), 100% FBS, and 100% human serum on the CS-ZI-AuNPs nanohydrogel surface, showing antifouling.


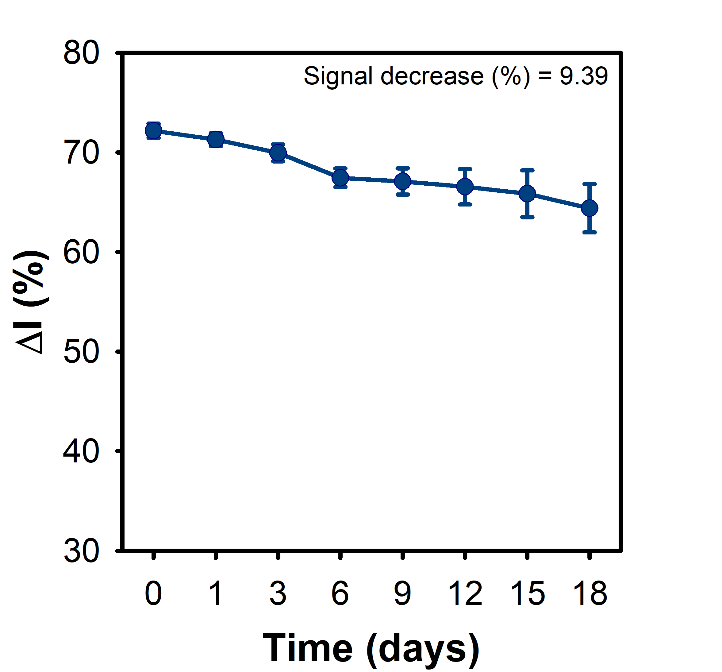


**Figure S7.** Stability test of the developed biosensor over 18 days.
